# Condoms as evidence of prostitution in the United States and the criminalization of sex work

**DOI:** 10.7448/IAS.16.1.18626

**Published:** 2013-05-24

**Authors:** Margaret H Wurth, Rebecca Schleifer, Megan McLemore, Katherine W Todrys, Joseph J Amon

**Affiliations:** Health and Human Rights Division, Human Rights Watch, New York, NY, USA

The vulnerability of sex workers and transgender women to HIV infection is a result of many factors including stigma, social and physical isolation, economic deprivation, and legal and policy environments that criminalize their behaviour [1,2]. Recent systematic reviews have found high HIV prevalence among both populations, including an 11.8% pooled HIV prevalence among female sex workers in 50 countries [3] and a 19.1% HIV prevalence among male-to-female transgender women in 15 countries worldwide [4]. Studies in the United States have also documented high HIV prevalence among people who report transactional sex [5] and transgender populations [6,7].

Among these diverse causes of vulnerability, a growing body of literature has examined the effects of the practice by police of using possession of condoms as evidence to support prostitution-related charges [1,8–12]. However, few studies have examined the health consequences of this practice in the United States. In New York City, research by the Department of Health and Mental Hygiene and the PROS Network (Providers and Resources Offering Services to Sex Workers) found that many sex workers reported having not carried condoms due to fear of police harassment [13,14]. Information from other US cities on police practices related to the use of condoms as evidence of prostitution, and the consequences on HIV-risk behaviours, has been limited.

Between October 2011 and July 2012, Human Rights Watch conducted mixed method research in four major US cities on the prevalence and consequences of police seizure of condoms as evidence of prostitution-related offenses, and the introduction of condoms as evidence of prostitution-related offenses in criminal proceedings. We interviewed nearly 200 current and former sex workers, as well as 110 outreach workers, advocates, lawyers, public defenders, prosecutors, judges, and public health officials in New York, Washington, DC, Los Angeles, and San Francisco. Documents on police and municipal health department practices were obtained through Freedom of Information Law and public record requests [15].

Our research indicated that in all four cities, police stopped, searched, and arrested people involved, or believed to be involved, in sex work using possession of condoms as evidence of intent to engage in prostitution-related offenses. Though few prostitution or loitering cases proceed to trial, prosecutors in New York, Los Angeles, and San Francisco have introduced condoms as evidence of prostitution-related offenses in criminal court.

The use of condoms as evidence of prostitution had the same effect in each of the four cities: while public health departments spend millions of dollars promoting and distributing condoms for HIV prevention, some members of the groups most at risk of infection – sex workers, transgender women, and lesbian, gay, bisexual, and transgender (LGBT) youth – fear carrying condoms to the point where they carry only a few, or none at all, and sometimes engage in sex without protection as a result [15]. Some women told Human Rights Watch that they continued to carry condoms despite the potential consequences. But for others, fear of arrest based upon possession of condoms overwhelmed their need to protect themselves from HIV, sexually transmitted infections, and pregnancy.

Sex workers, transgender women, and other LGBT people can face high levels of abuse, harassment, and violence in police custody and in prison [6,16,17]. For undocumented and documented migrants, arrest for prostitution-related offenses can mean detention and removal from the United States [18].

Change is possible. Since the publication of our research findings, the US Presidential Advisory Council on HIV/AIDS (PACHA) has adopted a resolution recognizing that the use of condom possession as evidence of prostitution-related offenses, like laws treating HIV status as the basis for prosecution or sentence enhancement, undermines HIV prevention and testing efforts. The Council urged the Departments of Justice and Health and Human Services to amend criminal laws and health policies to ensure consistency with medical and scientific knowledge and human rights [19]. District attorneys in two jurisdictions – Nassau County, NY, and San Francisco – have stopped using condoms as evidence of prostitution and police in Washington, DC, are distributing know-your-rights cards to affected populations clarifying that the Metropolitan Police Department cannot interfere with possession of condoms and providing information on how to file a complaint against an officer for harassing, stopping, or searching a person on the basis of carrying condoms. These programmes reflect collaboration between affected communities, law enforcement, and public health officials.

However, these initiatives do not go far enough. The criminalization of voluntary, consensual sexual relations among adults is incompatible with respect to a number of internationally recognized human rights [20]. Human Rights Watch has repeatedly documented the negative consequences of the criminalization of sex work on access to rights to personal autonomy and privacy, freedom from discrimination and violence, health, and justice [1,15,21–23].

At the same time, the decriminalization of sex work is controversial, and claims of a human rights foundation for decriminalization are contested. Some advocates believe that sex work is a form of sexual violence, while others argue that sex work represents the commodification for sale of the human person, which they view as incompatible with human dignity [24,25].

In our view, these positions that cast sex workers only as victims, and eliminate the possibility of voluntary sex work, deny sex workers’ agency and their rights to exercise their autonomy and engage in private, consensual behaviour. Gender inequality and poverty certainly are important factors that lead some individuals to participate in sex work. However, criminalizing sex work does not directly counter these problems [26,27]. The promotion of social and economic rights to counter discrimination based on grounds such as sex, sexual orientation or gender identity, race, ethnicity or immigration status, are important, existing requirements of international human rights law.

Criminalizing sex work contributes to conditions under which sex workers are reluctant to report violence and abuse by police and others for fear of incriminating themselves or provoking police retaliation. Although decriminalization would not eliminate all the risks inherent in sex work, it would allow sex workers to self-organize and work with law enforcement officials. Empowerment of sex workers through legal recognition of their occupation also maximizes their protection and dignity and helps to ensure equal access to health services and to justice [26,28].

Decriminalization is particularly important when simple HIV prevention measures, such as the possession of condoms, are used as evidence of a criminal act. While prohibiting the use of condoms as evidence of prostitution is an important first step, Human Rights Watch has concluded that ending the criminalization of sex work is critical to achieving public health and human rights goals.
